# Postgraduate nursing internationality education: A study in establishing an English nursing course

**DOI:** 10.1002/nop2.536

**Published:** 2020-06-22

**Authors:** Yong‐bing Liu, Wei‐juan Gong, Xin‐e Mao

**Affiliations:** ^1^ School of Nursing Yangzhou University Yangzhou China

**Keywords:** curriculum, education, graduate, internationality, nursing

## Abstract

**Aim:**

The study was to explore the significant influence of an English nursing course in nursing postgraduate internationalization education.

**Design:**

A cross‐sectional study.

**Method:**

The research object included three grades (a total of 18) of nursing postgraduate students in the Nursing School of Yangzhou University. A standardized four‐section questionnaire designed by the authors was applied to the survey.

**Results:**

88.89% were satisfied with the course design and application of the English nursing course, and the scores for all 6 items were above the average, but only 44.4% of the postgraduate students understood the course completely. In teaching design and content aspect, 88.9%–94.4% postgraduate nurses felt that the course learning requirements were clear and could improve their knowledge and capacity for scientific research. All of the postgraduate nurses were identified with the teacher's moral and academic characters.

**Conclusions:**

To establish English nursing courses for nursing postgraduate students is beneficial for postgraduate internationalization education.

## 1 INTRODUCTION

As the demand for more nurses with advanced degrees increases, so does enrolment in postgraduate nursing projects. Because of this, nursing schools of universities are improving their postgraduate programs, curricula and facilities to attract students interested in furthering their nursing education.

Postgraduate nursing education involves further study after completion of a bachelor's degree (Boore, 1996). In the nursing sciences field, postgraduate education generally recommends that a graduate specializes in a particular field of study within the nursing discipline. For most disciplines, this further study is at a postgraduate diploma or master's level (McInerney & Green‐Thompson, 2017).

Education of postgraduate medical student**s** is an important part of higher education in China (Guo, Liu, Li, Guo, & Wang, 2016). Educational reform has not only been expressed by the increase in number and categories of postgraduates, but also by a significant internationalization trend in nursing postgraduate education in China (Wang, 2008). Most nursing schools of universities offer quality postgraduate programs of English for specific purpose that provide students with opportunity to study a wide range of international nursing specialty courses.

The benefits of this international nursing course experience include professional, theoretical‐scientific improvement and the realization of social and cultural values. The contact with other people and educational institutions allows different didactic, pedagogical and interpersonal skills (Marcio, Rosa, & Maria‐Antonia, 2018). The kind of studying process enables the strengthening and recognition of China postgraduate educational system, encourages nursing postgraduate students to know different realities and thus strengthens the internationalization of nursing teaching and research.

## 2 BACKGROUND

To promote global competitiveness and standards, the China State Council issued a document (Overall Plan to Promote the World First‐class University & First‐class Discipline Construction) on 5 November 2015 (China State Council, 2015). The Chinese government has endeavoured to advance “Double First‐Rate” construction, with respect to the higher education policy; China will construct a batch of universities and disciplines into world first‐class universities and disciplines (Gao & Gong, 2016).

According to the national policy, universities have paid increasing attention to internationalization education in recent years. Higher education internationalization needs university leaders with an international perspective and a strong, multidisciplinary academic background. In particular, university leaders can regard internationalization as a major task to fulfil. Yangzhou University attaches more importance to internationalization of higher education, especially postgraduate education. Internationalized graduate education is not only an important symbol of higher education internationalization, but it is also a prominent feature of world‐class universities at home and abroad (Gao & Gong, 2016).

In addition to policy, there has been increasing development of an internationalized curriculum and promotion of international academic exchanges. English for specific purpose is the essence for developing academic research capacity, which is a core component for postgraduate education (Zhang, Jin, & Du, 2020). In the healthcare context, English language accounts for more than 80% of medical journals, databases, online resources and international academic conferences (Zhang, 2013). English language and academic research capacity are two known core components of postgraduate students in China. Skilled English language ability, especially, English for specific purpose, plays a crucial role in smoothing academic activities and thereby improving professional development and communication (Wang, 2010).

For example, one of the internationalizing measures of Yangzhou University is construction of a full English graduate curriculum. The graduate school set up several courses which will be taught in English every semester. These courses were selected by evaluation experts based on many course application materials submitted by various institutes. When the project is approved, the course will be supported by the graduate school with policy, funding and location requirements. The teachers can be invited from different countries and regions, including Hong Kong, Macao and Taiwan. The teachers should be well‐known experts, scholars or professors.

After several years of construction, the concept of English for specific purpose curriculum for postgraduate students should focus on strengthening the understanding of disciplinary knowledge in science and enhancing international academic communication ability through English language acquisition. When curriculum activities are designed, educational principles and theories should also be followed and integrated to ensure the efficacy of teaching and realize the win‐win situation of teachers and students (Zhang et al., 2020). In such a climate, an effective supply of English for specific purpose curriculum for nursing postgraduate students has become particularly important.

This year, the nursing school applied for a postgraduate English nursing course (Health Promotion and Nursing of Older Adults); the course has 2 credits and 36 class hours. The course is consistent with the ageing population in China. Throughout this course, nursing postgraduate students will learn about gerontologic nursing research progress, theoretical framework, health‐promoting theory and the nursing method for chronic diseases of older adults. We have invited two experts from the Nursing School of the Hong Kong Polytechnic University. The nursing core course is compulsory for the master's degree.

The study was designed to measure how the English nursing course affects the nursing postgraduate students from course design and application, teaching design and content, moral and academic characters of teachers and the expectation for further courses taught in English. This study is an important part of the project.

## 3 METHODS

### 3.1 Study design

This was a cross‐sectional study of baseline data. From October–November 2017, we obtained the evaluation data of nursing postgraduates to English nursing course. A self‐design standardized four‐section questionnaire was used in the research.

### 3.2 Setting and sample

The nursing postgraduates were recruited from the Nursing School of Yangzhou University. Our postgraduate educational system is 3 years in length. All the nursing postgraduates had academic degrees. Among the nursing postgraduates, eight were Grade one, five were Grade two, and five were Grade three (total = 18).

The inclusion criteria for the investigation were as follows: studying for a master's degree; selected the English nursing course; and willing to participate in the study after informed consent regarding the purpose and procedure. The exclusion criterion was failure to complete the course.

### 3.3 Ethical consideration

The study was approved by the Institutional Review Board of Yangzhou University (there are no IRB approval numbers). All postgraduates provided informed consent before the study started. All participating postgraduates were notified of the nature and objectives of the investigation. Confidentiality and anonymity were maintained throughout the entire study period.

### 3.4 Measurements and instruments

A standardized four‐section questionnaire designed by the authors was used for the study. The questionnaire contained 30‐items and included detailed questions about course design and application, teaching design and content, and the moral and academic characters of the teacher. Among the 30 items, there were 21 objective and 9 subjective problems. A 5‐point Likert scale was used for the objective problems, with scores ranging from 5 (strongly agree)–1 (strongly disagree). The Cronbach alpha coefficient of this questionnaire was 0.73, indicating good reliability.

### 3.5 Data collection and procedure

In this study, the researchers recruited nursing postgraduates who wished to participate in the study voluntarily by posting a recruitment notice on a classroom bulletin board. Once the nursing postgraduates were confirmed, the researchers obtained written consent from all nursing postgraduates. Data collection was conducted after the English nursing course was finished. Course design and application, teaching design and content, and the moral and academic characters of the teacher were measured using the questionnaire.

### 3.6 Data analysis

Descriptive statistics and non‐parametric analysis were calculated for categorical variables. The reliability of the questionnaire was tested for internal consistency using Cronbach's alpha coefficient. The database was established using Excel. Data were analysed using SPSS 18.0 software (SPSS, Inc., Chicago, IL, USA). Continuous variables are presented as the mean ± standard deviation (*SD*). Statistical significance was defined at values of *p < *.05.

## 4 RESULTS

### 4.1 Basic information

Postgraduate nurses had a mean age of 26.70 (*SD* 4.13) years. Research directions included clinical nursing, gerontologic nursing and nursing education.

### 4.2 Course design and application

The survey results for course design and application showed that the scores for 6 items were 3.28 (*SD* 0.67), 3.64 (*SD* 0.80), 3.81 (*SD* 0.61), 3.56 (*SD* 0.76), 4.10 (*SD* 0.84) and 4.27 (*SD* 0.70); the scores for all 6 items were above the average. All questions had highly statistically significant differences (*p *< .001 [two‐tailed]). Of the postgraduate students, 77.8% thought that the English nursing course could have an impact on their graduate study period; 88.9% thought that the English nursing course could enlighten their future road of scientific research; 61.1% agreed that the course has an important influence to understand scientific research methods; 83.4% agreed that they were interested in the course; and 88.9% assessed the establishment of an English nursing course as satisfactory. Only 44.4% of the postgraduate students understood the course completely based on their facility with English. The results of the questionnaire are summarized in Table 1.

**TABLE 1:** Frequency of postgraduate student responses to course design and application questions [score and number (%)]

Item	Scores	Number (%)						
		Strongly agree	Agree	Uncertain	Disagree	Strongly disagree	*ᵡ^2^*	*p*
You can understand the course	3.28 ± 0.67	0 (0.0)	8 (44.4)	9 (50.0)	1 (5.6)	0 (0.0)	24.64	<.001
Impact on your graduate study period	3.64 ± 0.80	4 (22.2)	10 (55.6)	3 (16.6)	1 (5.6)	0 (0.0)	30.73	<.001
Enlighten your future road of scientific research	3.81 ± 0.61	3 (16.7)	13 (72.2)	2 (11.1)	0 (0.0)	0 (0.0)	30.09	<.001
Have an important influence to understand scientific research methods	3.56 ± 0.76	3 (16.7)	8 (44.4)	7 (38.9)	0 (0.0)	0 (0.0)	33.72	<.001
Your interest in the course	4.10 ± 0.84	10 (55.6)	5 (27.8)	3 (16.6)	0 (0.0)	0 (0.0)	32.87	<.001
Your assessment of the course design	4.27 ± 0.70	12 (66.7)	4 (22.2)	2 (11.1)	0 (0.0)	0 (0.0)	31.20	<.001

### 4.3 Teaching design and content

The survey results of teaching design and content showed that the scores of nine items were 4.64 (*SD* 0.48), 4.79 (*SD* 0.49), 4.31 (*SD* 0.82), 4.58 (*SD* 0.50), 4.62 (*SD* 0.60), 4.53 (*SD* 0.59), 4.58 (*SD* 0.61), 4.65 (*SD* 0.48) and 4.24 (*SD* 0.65); the scores for all 9 items were above the average. All the questions showed highly statistically significant differences (*p *< .001 [two‐tailed]). Of the postgraduate nurses, 88.9%–94.4% felt that the course learning requirements were clear, the textbooks were of high quality, and the teaching information was moderated, captured the latest international trends and helped students to improve their knowledge and capacity for scientific research. The results of the questionnaire are summarized in Table 2.

**TABLE 2:** Frequency of postgraduate student responses to teaching design and content questions [score and number (%)]

Item	Scores	Number (%)						
		Strongly agree	Agree	Uncertain	Disagree	Strongly disagree	*ᵡ^2^*	*p*
Course learning requirements clearly (including teaching objectives and assessment methods)	4.64 ± 0.48	11 (61.1)	6 (33.3)	1 (5.6)	0 (0.0)	0 (0.0)	23.64	<.001
High quality textbooks or handouts (including rich references and bibliography)	4.79 ± 0.49	14 (77.8)	3 (16.7)	1 (5.6)	0 (0.0)	0 (0.0)	19.65	<.001
Moderated teaching information, combined theory, and practice	4.31 ± 0.82	8 (44.4)	8 (44.4)	1 (5.6)	1 (5.6)	0 (0.0)	10.22	.007
Seize the latest international tendency of gerontologic nursing	4.58 ± 0.50	10 (55.6)	7 (38.8)	1 (5.6)	0 (0.0)	0 (0.0)	24.31	<.001
Thinking clearly, teaching vividly, and stimulating interest	4.62 ± 0.60	12 (66.7)	5 (27.7)	1 (5.6)	0 (0.0)	0 (0.0)	22.64	<.001
To expound academic thought and research methods precisely	4.53 ± 0.59	10 (55.6)	7 (38.8)	1 (5.6)	0 (0.0)	0 (0.0)	24.31	<.001
Enlightening teaching and encouraging students to think independently and innovatively	4.58 ± 0.61	11 (61.1)	6 (33.3)	1 (5.6)	0 (0.0)	0 (0.0)	23.64	<.001
Diversified teaching form and methods, and improved effective teaching	4.65 ± 0.48	11 (61.1)	6 (33.3)	1 (5.6)	0 (0.0)	0 (0.0)	23.64	<.001
Helpful for students to improve their fund of knowledge and scientific research	4.24 ± 0.65	6 (33.3)	10 (55.6)	2 (11.1)	0 (0.0)	0 (0.0)	24.66	<.001

### 4.4 Moral and academic characters of teachers

The survey results pertaining to the moral and academic characters of teachers showed that the scores of 6 items were 5.00 (*SD* 0.00), 4.81 (*SD* 0.39), 4.94 (*SD* 0.24), 4.79 (*SD* 0.41), 4.95 (*SD* 0.21) and 4.61 (*SD* 0.30); the scores for all 6 items were very high; all of the nursing postgraduates were identified with the moral and academic characters of teachers. The results of the questionnaire are summarized in Table 3.

**TABLE 3:** Frequency of postgraduate student responses to the academic characteristics of teacher questions [score and number (%)]

Item	Scores	Number (%)				
		Strongly agree	Agree	Uncertain	Disagree	Strongly disagree
The teacher is strict and prepares lessons adequately	5.00 ± 0.00	18 (100.0)	0 (0.0)	0 (0.0)	0 (0.0)	0 (0.0)
Be strict with the learning and research abilities of students	4.81 ± 0.39	14 (77.8)	4 (22.2)	0 (0.0)	0 (0.0)	0 (0.0)
Patiently communicate with students both inside and outside the class	4.94 ± 0.24	17 (94.4)	1 (5.6)	0 (0.0)	0 (0.0)	0 (0.0)
Make good use of the current frontier knowledge	4.79 ± 0.41	14 (77.8)	4 (22.2)	0 (0.0)	0 (0.0)	0 (0.0)
Have profound academic attainments and their own teaching style	4.95 ± 021	17 (94.4)	1 (5.6)	0 (0.0)	0 (0.0)	0 (0.0)
Overall view of teachers is satisfied	4.61 ± 0.30	15 (83.3)	3 (16.7)	0 (0.0)	0 (0.0)	0 (0.0)

## 5 DISCUSSION

Postgraduate education is key to improving the knowledge, skills, attitudes and competence of professional nurses (Estrada‐Masllorens et al., 2016). Establishing English nursing courses was our first attempt in developing postgraduate education for the Nursing School at Yangzhou University. Our nursing postgraduate students were satisfied with the English nursing course. The course enlightened scientific research thinking and broadened the international perspective of the students. In general, the English nursing course achieved the established target.

With respect to course design and application, the results demonstrated that nursing postgraduates were highly satisfied with the course design and application; the scores of the six items were all above average. All questions showed highly statistically significant differences (*p *< .001 [two‐tailed]). Of the nursing postgraduates, 78% thought that the English nursing course could have an impact on their graduate study period; 88.9% thought that the English nursing course could enlighten their future road of scientific research; 83.4% agreed that they were interested in the course; and 88.9% assessed the establishment of an English nursing course as satisfactory.

We have found that our postgraduate nurses do not have adequate preparation to face the internationalization education. Only 44.4% of the postgraduate nurses thought that they could master the course content completely. Their level of expressive English and applied competence required further improvement, especially in the professional English terms used in the communication of classroom discussion. Next, we strengthened the recognition degree of postgraduate nurses to the study of the English language. Ultimately, postgraduate nurses rapidly blend into internationalization education.

In general, holders of master's degrees in nursing should have more in‐depth specialized knowledge and a greater capacity to think about scientific research (Palucci‐Marziale & Garcia de Lima, 2015). They are qualified to assume the responsibility of research. The English nursing course design and application were consistent with postgraduate demand.

The results indicate that postgraduate nurses approved of the teaching design and content. Of the postgraduate nurses, 88.9%–94.4% felt that the course learning requirements were clear, captured the latest international trends and helped students to improve their knowledge and capacity for scientific research.

A teaching strategy designed to expose students to practical contexts significantly enhanced their research skills (Xu, Dinwoodie, & Chang, 2012). Griffiths (2004) defined four approaches to implement the teaching–research nexus. In “research‐oriented” teaching, the method offers students the opportunity to learn how to undertake research within their discipline. Teachers instruct students regarding enquiry skills, rather than merely presenting research findings. The curriculum is structured on an understanding of research process and methods, rather than subject content. Our postgraduate students felt that the course improved their level of knowledge and capacity for scientific research.

All of the postgraduate nurses were identified with the moral and academic characters of the teacher. In MacIntyre's view, both the integrity of the practice and the good functioning of the institutions depend on the moral character of individuals, that is, their exercise of moral agency vis‐à‐vis their identity and social role (Pianezzi, Norreklit, & Cinquini, 2019). It is very important that the teachers should have excellent moral and academic characters when they teach the English for specific purpose curriculum. One of the most important aims of schools is to develop student's character, intellectually and morally through value‐based atmosphere to be good moral members in their communities (Omar, 2018). Ministry of Education of China issued a document (Opinions on the full implementation the tutors duties to strengthening moral education of postgraduate) on 18 January 2018; one of the basic qualities of tutors is noble morality.

According to the Yangzhou University document requirements, we should focus on well‐recognized experts, scholars and professors. In addition, considering the development of the postgraduate nurses' career, the teacher should possess moral nobility and profound knowledge.

With respect to the nine subjective problems, we wanted to understand the feelings of the postgraduate nurses regarding the English nursing course; the domestic nursing teaching system compared with Hong Kong; and further requirements for the English course.

Most of the postgraduate students characterized the expert as rigorous with a practical research attitude and novel teaching method, combined with clinical practice, holistic nursing concept and an internationalization view.

In addition, the postgraduate students hope to construct more English nursing courses, such as nursing research, nursing psychology, rehabilitation nursing, community care, clinical nursing and nursing management. The postgraduate students are interested in additional opportunities to communicate with the world.

The current research had some limitations. Firstly, the sample size was small with only 18 postgraduate students, and the results may not have enough persuasion. Secondly, combining with qualitative and quantitative research design methods will reflect the thought of nursing postgraduate students comprehensively; we only adopted quantitative research and lost the opportunities to mining problems deeply. Finally, due to the limitation of English listening and expression, the learning effect of some nursing postgraduate students is affected to some extent.

The findings highlight further research into ways to improve nursing postgraduate education internationalization. The next step in research for nursing internationalization education is to improve the standards of English language among postgraduate nurses and expand the English nursing course in depth and breadth. Most of the postgraduate students indicated that they want more in‐depth research training to improve their competency. In addition, the postgraduate students want to obtain more professional knowledge on the forefront of their nursing discipline.

In the future, according to the different requirements of nursing postgraduate students, we will offer appropriate courses of English for specific purpose, to meet their needs of desiring to obtain the nursing education of internationalization. Firstly, we can introduce research‐based learning into nursing courses of English for specific purpose, which is consistent with educational philosophy of Yangzhou University. During the nursing courses of English for specific purpose, the teachers initiate nursing postgraduate students' spirit of curiosity, exploration and scientific research and improve language acquisition and professional technical abilities by collaboration and interaction between teachers and students. Secondly, we will develop the integrated curriculum, which reflects the combination of English language capabilities and interdisciplinary integration, expanding language studying to various domains of humanities and social sciences, thus allowing students to discover and understand the difference between nursing disciplines and relative fields of the world through topic research based on life experience. Through learning of integrated curriculum, nursing postgraduate students' potential and existing knowledge will be inspired and interpersonal skills and comprehensive quality will be promoted. Those components enable students to naturally learn other subjects and provide boost to their professional career.

## 6 CONCLUSION

This study demonstrated that nursing postgraduate students welcome an English nursing course. An English nursing course can broaden their international perspective, experience different teaching styles, study international groundbreaking ideas and improve nursing research competency. In the future, we should construct more English nursing courses to perfect their knowledge base. These beneficial endeavours will play an important role in nursing postgraduate internationalization education.

## CONFLICT OF INTEREST

The authors declare no conflict of interest.

## AUTHOR CONTRIBUTIONS

Yong‐bing Liu: Study design, study conduction, collection and analysis of data, and manuscript draft preparation. Wei‐juan Gong: Data collection, statistical analysis and manuscript draft preparation. Xin‐e Mao: Data collection. All authors read and approved the final manuscript.
